# Minimally Invasive Management of Hemorrhagic Pheochromocytoma—A Rare Case Report

**DOI:** 10.1055/s-0043-1762554

**Published:** 2023-03-26

**Authors:** Ajay H. Bhandarwar, Amarjeet E. Tandur, Keerthika Reddy Rachapalli, Amol Wagh, Abhijit Shah, Nikhil Dhimole

**Affiliations:** 1 Department of General Surgery, Grant Government Medical College and Sir JJ Group of Hospitals, Mumbai, Maharashtra, India

**Keywords:** hemorrhagic pheochromocytoma, minimally invasive surgery, rare complication, case report

## Abstract

Pheochromocytoma is a rare catecholamine-secreting tumor derived from chromaffin cells. The diagnosis is usually suggested by its classic history, presence of a strong family history, or discovery of an incidental mass on imaging in an asymptomatic patient. Hemorrhage into an occult pheochromocytoma is a rare complication with ∼1 to 2 per 100,000 individuals diagnosed annually. We report a case of a 29-year-old woman, who presented with abdominal pain (with no other significant history) due to a right hemorrhagic pheochromocytoma. Computed tomographic imaging and magnetic resonance imaging revealed the source of retroperitoneal hemorrhage as the right adrenal mass. They lacked the typical features of a pheochromocytoma which was eventually proven by the biochemical tests. The patient underwent preoperative stabilization with α and β adrenergic receptor blockers for 7 days following which laparoscopic adrenalectomy was performed successfully with an uneventful postoperative period. This is the eighth reported case in literature managed laparoscopically. Histopathology confirmed it as pheochromocytoma. The treacherous and deceptive nature of pheochromocytomas and its hemorrhage make it crucial to detect and treat it promptly; otherwise, it will almost certainly be fatal from cardiovascular complications or metastasis.

Pheochromocytoma is a rare catecholamine-secreting tumor of the adrenal glands presenting with a myriad of clinical and imaging manifestations. It can lead to over whelming cardiovascular crises if left undiagnosed or if appropriate treatment is delayed. 1 Acute onset of abdominal pain and nausea may be the only presenting symptoms of spontaneous hemorrhage in a pheochromocytoma—a rare and highly lethal complication with atypical imaging findings, making its diagnosis challenging. 2 We report a case with spontaneous rupture of the right adrenal pheochromocytoma presenting with abdominal pain in a 29-year-old woman with no history of hypertension, paroxysmal headaches, or palpitations.

## Case Report



**Video 1** Hemorrhagic pheochromocytoma.


**Fig. 1:**
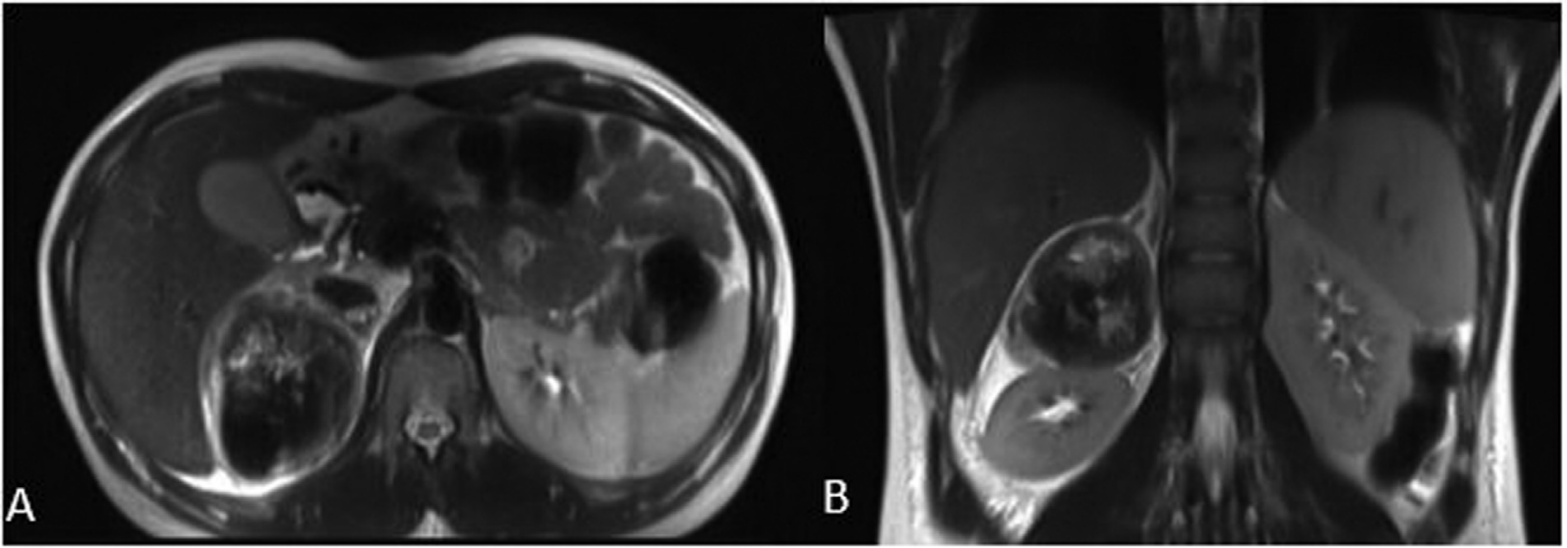
(A) Axial view and (B) coronal view of magnetic resonance imaging T2-weighted images showing a well-defined heterogenous hypointense lesion in the lateral limb of right adrenal gland.

**Fig. 2:**
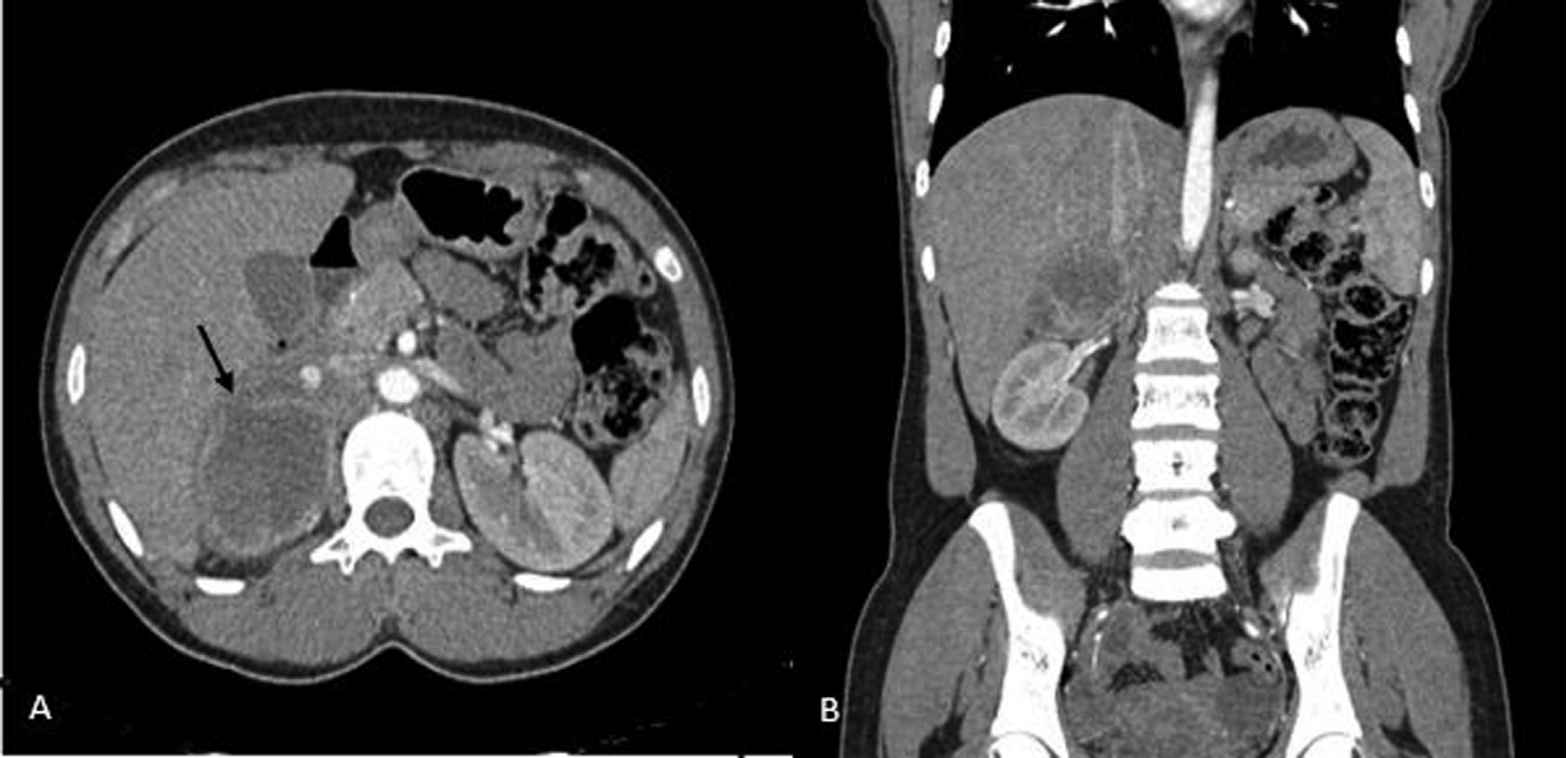
(A) Axial view and (B) coronal view of contrast-enhanced computed tomography showing a mass arising from the lateral limb of the right adrenal gland showing central liquefaction and intralesional hemorrhage with rupture (arrow) of the anterior wall leading to a walled off fluid collection extending into the anterior pararenal and subhepatic space.

A 29-year-old female kabaddi athlete visited the outpatient department due to progressive upper abdominal pain for the past 4 months with no other complaints, no history of steroid use in the past, and no comorbidities of note. Clinical examination was unremarkable except for tachycardia. She was further evaluated with an ultrasonography of the abdomen and pelvis which was suggestive of a large right adrenal mass composed of hypoechoic fat with cystic areas and mild surrounding fluid extending into the right perinephric space and paracolic gutter. These findings being suggestive of a ruptured adrenal myelolipoma with surrounding hemorrhage, a magnetic resonance imaging (MRI) of the abdomen was done for further evaluation and characterization. It showed a well-defined heterogenous lesion in the lateral limb of right adrenal gland ∼6.7 × 5.9 × 6.4 cm in size, hypointense on T1-weighted (T1W) and T2-weighted (T2W) sequences. No loss of signal is seen in the T1W out of phase images, representing no fat within the lesion with moderate free fluid in the right subhepatic, paracolic, and perinephric space suggestive of a hemorrhagic right adrenal adenoma ( Fig. 1 ). A coagulation profile and anticardiolipin antibody testing was further done (to rule out coagulation disorders and antiphospholipid antibody [APLA] syndrome) which were within normal range. Contrast-enhanced computed tomography (CT) of the abdomen showed a lesion likely arising from the lateral limb of the right adrenal gland showing central liquefaction and intralesional hemorrhage with rupture of the anterior wall leading to a walled off fluid collection extending into the anterior pararenal and subhepatic space. The lesion showed a 60.2% absolute washout on delayed images. This mass was suspected to most likely be a lipid poor adenoma or a pheochromocytoma ( Fig. 2 ).

Accordingly, blood and 24-hour collection of urine for metanephrine were ordered. It returned with a marked elevation of plasma-free metanephrine (109 pg/mL, normal <65 pg/mL), 24 hours urine metanephrine (833 µg/24 hours, normal <350 µg/24 hours) and metanephrine to creatinine ratio (453.92 µg/g creatinine, normal 33–109) confirming the diagnosis of pheochromocytoma.

**Fig. 3:**
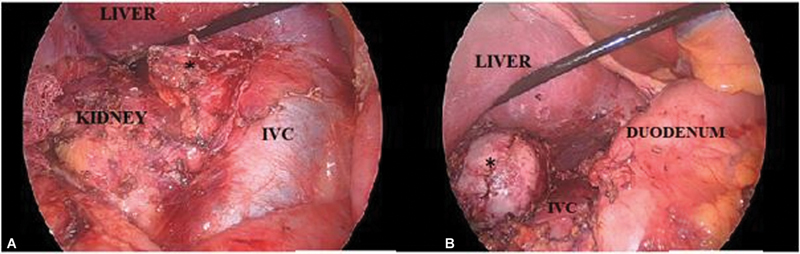
(A) Intraoperative image showing an enlarged right suprarenal mass (*) with contained retroperitoneal hemorrhage. (B) Completely dissected pheochromocytoma. IVC, inferior vena cava.

**Fig. 4:**
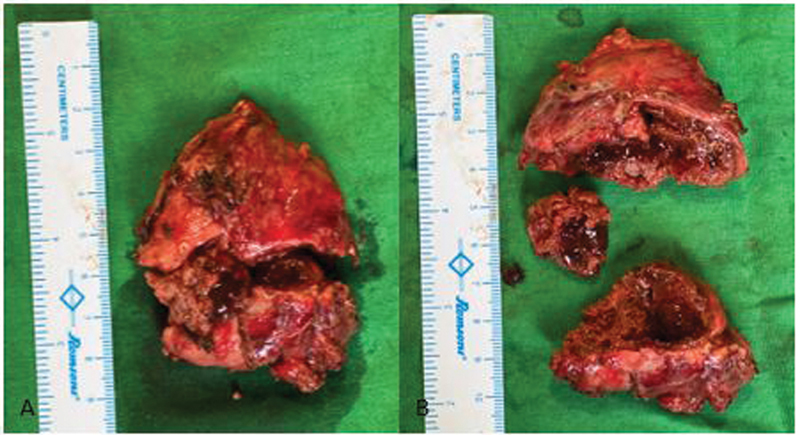
(A) Gross image of resected right adrenal mass. (B) Gross image of a cut open right adrenal mass showed variegated, partially cystic, and hemorrhagic cut surface.

The patient was optimized with preoperative prazosin (α-1 receptor blocker) and metoprolol (selective β-1 receptor blocker) and fluid loading for 7 days. Thereafter, laparoscopic right adrenalectomy was performed successfully. Intraoperatively, the right adrenal gland was identified with dense adhesions around the hilum due to hemorrhage and subsequent fibrosis and with a contained retroperitoneal hemorrhage ( Fig. 3 ). As the hilum was frozen, inferior dissection was done. An aberrant vein draining into the renal vein was identified. The rest of the hilar structures could not be identified due to the adhesions and was sheared with advanced bipolar device ( Video 1 ). During surgery, blood pressure was well controlled, although a few episodic rises required intravenous nitroglycerin. The total operative time was 3 hours ( Fig. 4 ).

The patient's blood pressure was within normal range in the postoperative period eliminating the need for any antihypertensives. She was discharged on postoperative day 3.

**Fig. 5:**
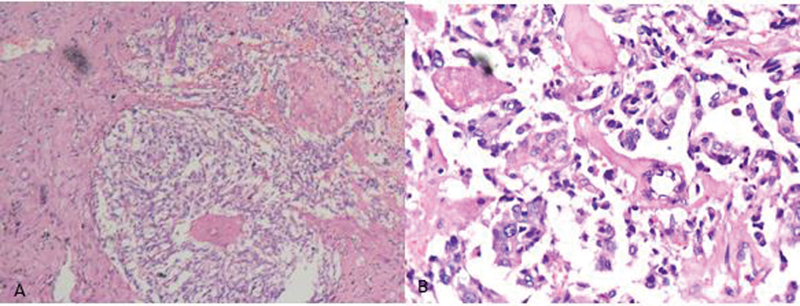
Hematoxylin and eosin staining (A) shows a highly cellular tumor arranged in a diffuse form with focal zellballen pattern and focal areas of necrosis and hemorrhage (×100 magnification). (B) Individual tumor cells have moderate eosinophilic to clear cytoplasm, round to oval vesicular. nuclei with inconspicuous nucleoli. Nuclear pleomorphism is present with tumor giant cells (×400 magnification).

**Fig. 6:**
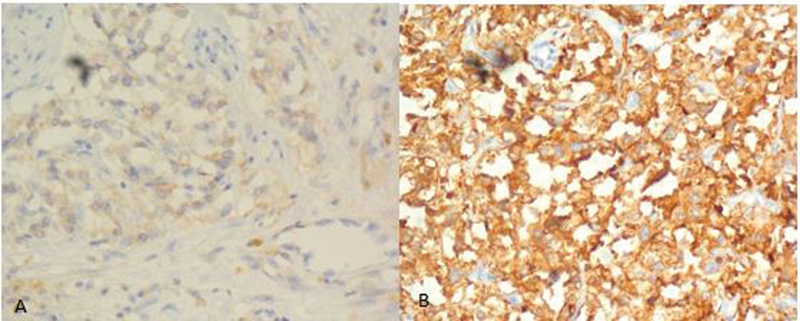
Positive staining for (A) chromogranin-A and (B) synaptophysin.

Histopathological evaluation confirmed it as a PASS score (Pheochromocytoma of the Adrenal gland Scaled Score) of 2 and MIB1 proliferative index of 8% at the margin suggestive of a benign tumor ( Fig. 5 ). Immunohistochemistry showed positive for chromogranin A and synaptophysin ( Fig. 6 ).

Genetic testing ruled out suspected germline mutations. Patient has been advised about continued annual endocrinology follow-up with biochemical tests.

## Discussion

Pheochromocytomas are rare, catecholamine secreting tumors with ∼1 to 2 per 100,000 individuals diagnosed annually. 3 They are composed of chromaffin cells of the adrenal medulla derived from embryonic neural crest cells which were first described by Frankel 4 in 1886 in a young woman likely afflicted with Multiple Endocrine Neoplasia type 2. 5 They may be either sporadic or a manifestation of hereditary (familial) syndromes, which are transmitted in an autosomal dominant fashion. 6

The key to diagnosing pheochromocytoma is to think of it first. Often referred to as “The Great Masquerader” 7 as it mimics various conditions, there is no single clinical finding that has significant value in diagnosing or excluding pheochromocytoma. 8 In two recent meta-analyses, 8 9 the symptoms with the greatest “pooled sensitivity” were hypertension (80.7%), headache (60.4%), palpitation (59.3%), and diaphoresis (52.4%). Pallor, nausea, flushing, anxiety or a sense of doom, palpitations, and abdominal pain can be part of the constellation of presenting symptoms. 10 It can rarely appear as “pheochromocytoma crisis,” a life-threatening condition 11 which presents with severe hypertension to circulatory failure and shock with subsequent involvement of multiple organ systems, including the cardiovascular, pulmonary, neurological, gastrointestinal, renal, hepatic, and metabolic systems. 11 Rarely, 1% is asymptomatic, representing an incidental discovery.

Several biochemical assays are available to facilitate diagnosis; however, plasma-free metanephrines have had the highest sensitivity and specificity in a recent multicenter cohort trial 12 in the detection of pheochromocytomas.

Once biochemical evidence of pheochromocytoma is obtained, imaging for localization should be undertaken to guide surgical resection ( Table 1 ).

**Table 1:** Different radiological modalities used in localizing a pheochromocytoma and paraganglioma and their typical imaging findings

1	Computed tomography	- First choice - Excellent special resolution for thorax, abdomen, and pelvis	- Solid/cystic, homogenous ± calcification - >10 HU - High attention due to hemorrhage - <50% washout
2	Magnetic resonance imaging	- In patients with metastatic pheochromocytoma - Detection of skull base and neck paraganglioma - Patients with surgical clips - Allergy to CT contrast - Where radiation exposure is to be limited (children, pregnancy, germline mutation)	- Low intensity at T1W imaging - High intensity at T2W imaging—light bulb appearance - Salt and pepper appearance on T1W and T2W in paraganglioma
3	123I-MIBG scintigraphy	- Patients with metastatic pheochromocytoma - When radiotherapy using 123I-MIBG is planned - Large sized tumor - Extra adrenal/multifocal/recurrent disease	- MIBG, octreotide, FDG+
4	FDG-PET	- Preferred over 123I-HIBG in patients with known metastatic pheochromocytoma	

Abbreviations: CT, computed tomography; FDG, fluorodeoxyglucose; MIBG, metaiodobenzylguanidine; PET, positron emission tomography; T1W, T1-weighted; T2W, T2-weighted.

Spontaneous rupture of pheochromocytoma is a rare event, with only ∼83 cases reported in the literature. Its presentation being highly varied, ranging from nothing more than nausea and abdominal pain to hemodynamic shock and abdominal catastrophe, resulting in delayed diagnosis. The exact mechanism of pheochromocytoma rupture remains unknown. A massive release of catecholamines is probably associated with vasoconstriction in the tumor and subsequent necrosis and hemorrhage. Consequently, elevated intracapsular pressure may result in a tear in the capsule, causing further hemorrhage into the retroperitoneum. 13 Trauma, 14 medications, 15 16 coagulation disorders, and APLA syndrome have also been implicated in hemorrhagic complications.

Clinical diagnosis of a ruptured pheochromocytoma is difficult and only 30.2% of patients have been diagnosed preoperatively 17 due to the following reasons. First, metaiodobenzylguanidine (MIBG) and F-18-fluorodeoxyglucose do not accumulate to the mass with total coagulation necrosis. Second, previous case reports for ruptured pheochromocytoma often failed in proving elevated levels of catecholamines because of difficulty in performing hormonal examination under emergency situation 18 and early normalization in plasma and urinary levels of catecholamines. 18 19 Third, in the diagnosis process of the ruptured pheochromocytoma, there is a strong correlation between hemodynamic instability and rate of misdiagnosis. 18 20

In our experience with laparoscopic adrenalectomy, this is the first encounter with a ruptured hemorrhagic pheochromocytoma and the eighth documented case in literature managed laparoscopically. 21 In this case, we faced a unique set of circumstances. CT scanning is the diagnostic tool of choice, but the lesion in our case showed an absolute washout of ∼60.2% as opposed to a distinctive <50% washout seen in a classical pheochromocytoma, thereby suggesting the possibility of it being a lipid poor adenoma. The lesion also failed to show a characteristic hyperintensity on T2W images on MRI, giving the impression of a ruptured adrenal hematoma. Hence, APLA syndrome (with a normal β2-cardiolipin levels) and a history of steroid usage was ruled out. Pheochromocytoma can undergo a variety of pathological degenerations, which affect their imaging features. This varied and changeable appearance merits the “chameleon” epithet given to this tumor. Plasma-free and 24 hours urinary metanephrines were tested which were elevated confirming the diagnosis of pheochromocytoma.

The treatment and outcome of the 83 cases are summarized in Table 2 . Mortality rate can be as high as 38.7% in patients with emergency adrenalectomy. However, there has only been 1 fatality (2.9%) reported among the 10 patients who underwent delayed surgery with transangiographic embolization (TAE).

**Table 2:** Summary of treatment, outcome and mortality rate of the 83 cases of ruptured pheochromocytoma

Treatment	Emergency surgery 36 (43.3%) Elective surgery 24 (28.9%) Conservative surgery 13 (15.6%) Elective surgery after TAE 10 (12%)
Outcome	Survived 63 (75.9%) Died 20 (24.0%)
Mortality rate	Emergency surgery or conservative treatment: 38.7% Delayed with or without TAE: 2.9%

Abbreviation: TAE, transangiographic embolization.

Urgent surgery is not indicated even in the presence of hemorrhagic pheochromocytoma because it has been shown that delayed surgery is associated with fewer intraoperative and postoperative complications and also lower mortality rate. 22 In the setting of a contained hematoma (like in this case), every effort should be taken to avoid emergency surgical intervention. Adequate medical preparation results in a mortality rate similar to that observed for elective adrenalectomy in the absence of hemorrhage. Medical optimization should include adequate blood resuscitation, correction of any coagulopathy to limit continued hemorrhage, hemodynamic support as needed, and ultimately α-blockade followed by volume expansion and β-blockade in an inpatient setting. Emergent surgical intervention may be considered in cases refractory to maximal medical management as recently described by May et al 14 with recognition of the high morbidity and mortality. In the absence of adequate adrenergic blockade in these extreme cases, the intraoperative and postoperative care must be tailored to the clinical picture as it evolves. Thus, the anesthesia and surgical teams must be prepared to manage sudden cardiovascular collapse, fulminant heart failure, massive pulmonary edema, and ongoing hemorrhage. If the tumor has been removed in toto, there is no need to continue antihypertensives postoperatively. Moreover, recent compelling data are emerging that in normotensive asymptomatic patients, preoperative α- blockade may not be necessary. 23 Although these data are provocative, they require validation from other centers, ideally in a prospective randomized fashion. It is the view of the committee that for such patients, preoperative blockade remains recommended to prevent unpredictable increases in blood pressure during surgery. 24

An alternative that has been receiving attention is TAE of the adrenal vessels which provides good control of ongoing hemorrhage in a patient who is unstable and unprepared for an exploration.

A large pheochromocytoma (>5 cm) warrants an ^123^ I-MIBG scan to look for metastasis. 25 The scan showed no metastasis. According to Endocrine Society Clinical Practice Guidelines, it is recommended that all patients with (pheochromocytomas and paragangliomas (PPGLs) should be engaged in a shared decision-making for genetic testing with a clinical feature-driven diagnostic algorithm to establish the priorities for specific genetic testing of suspected germline mutations. 26 Specifically, pretest and posttest counseling should be available. All tests for PPGL genetic testing should be performed by accredited laboratories. 26

## Conclusion

Spontaneous intraperitoneal hemorrhage remains a rare complication of pheochromocytoma, though the physiologic consequences present considerable medical and surgical challenges. Unfortunately, emerging clinical scenarios do not allow for optimal preoperative medical preparation with α-adrenergic blockade, and are associated with a high mortality rate. In the present case, as the patient was hemodynamically stable, she was optimized with antihypertensives preoperatively and managed through elective laparoscopic approach. Most importantly, a high index of suspicion must be maintained in similar cases so that the highly lethal hemodynamic sequelae may be anticipated and managed with the appropriate pharmacologic agents to ensure optimal outcomes. In unstable patients, a trial of TAE can be done.
